# Gaining Entrée into a Micronesian Islander-Based Community Organization Through Culturally Responsive Team Building and Reflection

**DOI:** 10.31372/20200503.1099

**Published:** 2020

**Authors:** S. Robert Spence Jr, Jacqueline Leung, Shelley Geil, Connie K. Y. Nguyen-Truong

**Affiliations:** a Washington State University College of Nursing in Vancouver, United States; b Micronesian Islander Community; c Dr. Connie Kim Yen Nguyen-Truong is the last senior author.

**Keywords:** Micronesian Islander, community organization, culturally responsive, team building, reflection, early childhood learning, adverse childhood experiences, community-academic partnership, sustainable

## Introduction

Building trust and rapport is crucial in developing sustainable relationships with communities of color who have suffered historical trauma (Nguyen-Truong, Closner, & Fritz, 2019; ^1^Nguyen-Truong, ^1^Leung, & Micky, 2020a). A history of nuclear weapons testing by the United States in Micronesia, and subsequent ill-prepared cleanup efforts, has created a historical trauma for the Micronesian Islander community (Letman, 2013). The purpose of this brief article is to describe a critical foundational engagement project approach when gaining entrée into a Micronesian Islander community-based organization to co-develop the culturally relevant main project to improve rates of Micronesian Islander enrollment in early childhood learning (ECL) programs. Building a sustainable community-academic partnership through culturally responsive team (CRT) building and leveraging the collective strengths, to address a community need, took half a year for relationship building, and shared decision-making.

## Problem/Significance of Topic

We describe the ECL need as background context. ECL is an important factor in preventing adverse childhood experiences (ACEs) and developing resilience from ACEs (Ellenbogen, Klein, & Wekerle, 2014; Oregon Health Authority, n.d.). ACEs are traumatic events experienced during childhood for example, psychological, physical, or sexual abuse; discrimination; and violence against a parent, particularly the mother (Felitti et al., 1998). Exposure to ACEs increases risk of adverse health outcomes including ischemic heart disease, cancer, obesity, and a myriad of psychosocial problems. Micronesian Islanders and other Asian/Pacific Islanders experience ACEs at rates higher than other ethnic groups (Kaholokula, Okamoto, & Yee, 2019). Although the data were not disaggregated into subgroups, Sieben, Lust, Crose, Renner, and Nguyen (2019) found that Asian/Pacific Islanders were 10% more likely than Whites to witness parental physical abuse and over 8% more likely to be victims of parental physical abuse. The culturally relevant main project of developing a toolkit to improve Micronesian Islander enrollment in ECL programs was developed to address this problem. However, in order to gain entrée into the Micronesian Islander Community ogranization and co-develop the main project, CRT team dynamics must be considered. Previous researchers have reported ethical issues surrounding collaborative activities, including needing additional time, financial burden, uncomfortable feelings related to power-sharing, and disempowerment among ethnic minoritized groups (Hoekstra et al., 2020).

## Methods

Our team is culturally diverse, interdisciplinary, and cross-sectoral. The Health and Education Program team is an alliance between the Executive Director/certified community health worker with a Juris Doctorate (community researcher) at the Micronesian Islander Community nonprofit organization and the academic PhD nurse scientist (Vietnamese, community leader) at Washington State University College of Nursing since 2018. A Doctor of Nursing Practice Family Nurse Practitioner (DNP FNP) student (White non-Latinx) and the academic DNP Psychiatric Mental Health Nurse Practitioner (White non-Latinx) at Washington State University College of Nursing joined the team to build upon previous work (^1^Nguyen-Truong, ^1^Leung et al., 2020a; ^1^Nguyen-Truong, ^1^Leung et al., 2020b). The DNP FNP student led the engagement project approach. The Washington State University Office of Research Assurances has certified as exempt (#18011-001). We used tenets of popular education for building a sustainable team dynamic and to guide the culturally responsive interactions among team partners (Wiggins, 2012). Among the tenets are (1) creating an atmosphere of trust is important to foster sharing of ideas and experiences, (2) every person knows a great deal and to start with what people know, (3) knowledge from life experience is equal to that acquired through formal education, (4) knowledge develops through interactions among people, (5) to be active in own learning process, and (6) to organize action to facilitate real change. The foundational tenets of popular education align with both the team’s goals of the project and the work being completed in the project development sessions.

Six CRT building core exercises and team reflections were held as a part of the main project planning sessions from February to July 2020. See Table 1. Sessions were held in a face-to-face video conferencing format for flexibility as partners resided in different cities and social distancing during the COVID-19 pandemic. A Plus/Delta evaluation was completed on strengths and recommendations regarding co-learning (O’Connell & Vandas, 2015). The DNP FNP student collected field note based data on the process and observations of the interactions and verified through team discussions.

**Table 1:** Culturally Responsive Team Building Session Goals, Core Exercises, and Team Reflection

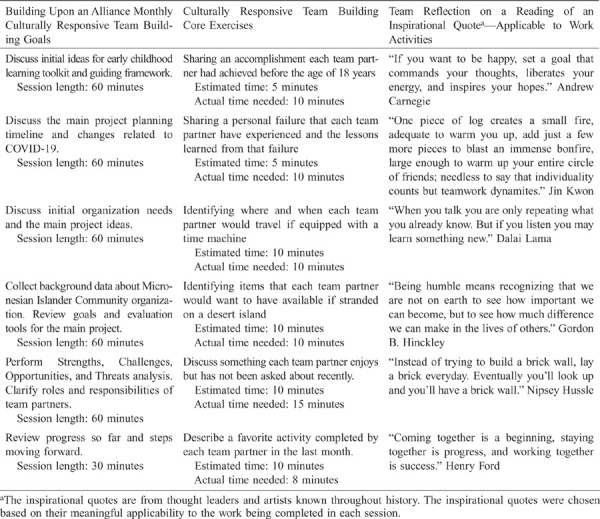

## Results

We identified three main themes. The first theme is inclusive leadership styles and the background that influenced partners in co-constructing a space for co-learning and shared decision-making. The team gained an appreciation for the contributions of viewpoints from partners. For example, in the exercise where partners selected items if stranded on a desert island, all gave different selections and reasons. While one partner chose a bag of oranges because they can provide nutrition, hydration, and a container for uses, another chose 100 feet of rope because of its versatility in use for situations including safely traversing treacherous terrain and building safe shelter. This exercise allowed partners to gain an understanding into each individual’s thought process and prioritization of needs. For example, regarding an evaluation tool, “The ECL toolkit will be used by the Micronesian Islander community” was changed to “I can use the ECL toolkit to assist in enrolling in ECL programs” in order to reflect both cultural appropriateness and clarity. The second theme is a dynamic balance and integration of cultural and worldview lens with a critical consciousness. The combined life experiences and cultural considerations provided a lens through which partners viewed both the problems at hand and the proposed main project interventions to address these problems. For example, one iteration of the proposed main project interventions included an entirely digital toolkit. The team decided that it would be necessary to include a print version with pictures due to inconsistent access to technology and language learning among Micronesian Islanders. The third theme is team reflection as an authentic journey. The team expressed increased motivation and had a greater appreciation and validation for the collective work. One example includes reflecting upon Kwon’s inspirational quote. The team expressed the contributions as a collective were greater than the sum of the individual work. For example, the Micronesian Islander Community organization shared about culturally sensitive implementation of teleconferencing due to COVID-19 to adapt methods of content delivery for the main project.

## Discussion

Developing an inclusive team dynamic that is culturally responsive is foundational in gaining entrée, building trust and relationships among stakeholders, and for co-designing the main project. The main project, development of a print and digital toolkit to assist with enrollment in ECL programs, will be implemented by the team in late Winter/early Spring 2021 to be used by Micronesian Islanders during the enrollment season. The identified themes underscore a transformative team dynamic where partners expressed an atmosphere of safety, expanded interpersonal knowledge, communication, and cultural understandings as a team and the Micronesian Islander community being served. Team partners felt free to share ideas and thoughts, and to learn from one another, without judgment or shame.

## Recommendations

CRT building core exercises and team reflection should be considered as potential means for gaining entrée into a new team dynamic and Micronesian Islander community organization, especially when that team consists of multicultural and interdisciplinary backgrounds. Our engagement project approach is translatable to other partnerships for sustainability of the partnership and the project. We recommend that this approach can be useful in gaining entrée to other diverse community organizations including other Pacific Islander and Asian communities.

## Acknowledgments

The authors are appreciative of the anonymous peer reviewers for assistance.

## Funding

The project was supported in part from the following grant funding. Dr. Connie Kim Yen Nguyen-Truong, PhD, RN, Alumnus PCCN, Doctor of Nursing Practice Family Nurse Practitioner Student Scholar S. Robert Spence, BSN, RN, and Dr. Jacqueline Leung, JD, MS, CHW, received the Delta Chi Chapter-At-Large Sigma Theta Tau International Scholar Award. Dr. Leung and Dr. Nguyen-Truong received the Health and Education Fund Impact Partnerships #19-02778, including Northwest Health Foundation, Meyer Memorial Trust, Kaiser Permanente Northwest, Care Oregon, and Oregon Community Foundation, and also the Asian Pacific American Network of Oregon through the Wallace H. Coulter Foundation.
